# Migration-prone glioma cells show curcumin resistance associated with enhanced expression of miR-21 and invasion/anti-apoptosis-related proteins

**DOI:** 10.18632/oncotarget.6092

**Published:** 2015-10-12

**Authors:** Wei-Lan Yeh, Hsiao-Yun Lin, Chiung-Yin Huang, Bor-Ren Huang, Chingju Lin, Dah-Yuu Lu, Kuo-Chen Wei

**Affiliations:** ^1^ Department of Cell and Tissue Engineering, Changhua Christian Hospital, Changhua, Taiwan; ^2^ Graduate Institute of Neural and Cognitive Sciences, China Medical University, Taichung, Taiwan; ^3^ Department of Neurosurgery, Chang Gung Memorial Hospital, Chang Gung University College of Medicine, Taoyuan, Taiwan; ^4^ School of Medicine, Tzu Chi University, Hualien, Taiwan; ^5^ Department of Neurosurgery, Taichung Tzu Chi General Hospital, Taichung, Taiwan; ^6^ Department of Physiology, School of Medicine, China Medical University, Taichung, Taiwan; ^7^ Department of Photonics and Communication Engineering, Asia University, Taichung, Taiwan

**Keywords:** migration, glioma, curcumin, miR-21, death receptor

## Abstract

In study, the expression patterns and functional differences between an original glioma cell population (U251 and U87) and sublines (U251-P10, U87-P10) that were selected to be migration-prone were investigated. The expressions levels of VEGF and intracellular adhesion molecule-1 (ICAM-1) were increased in the migration-prone sublines as well as in samples from patients with high-grade glioma when compared to those with low-grade glioma. In addition, cells of the migration-prone sublines showed increased expression of the oncogenic microRNA. miR-21, which was also associated with more advanced clinical pathological stages in the patient tissue specimens. Treatment of U251 cells with an miR-21 mimic dramatically enhanced the migratory activity and expression of anti-apoptotic proteins. Furthermore, treatment with curcumin decreased the miR-21 level and anti-apoptotic protein expression, and increased the expression of pro-apoptosis proteins and microtubule-associated protein light chain 3-II (LC3-II) in U251 cells. The migration-prone sublines showed decreased induction of cell death markers in response to curcumin treatment. Finally, U251-P10 cells showed resistance against curcumin treatment. These results suggest that miR-21 is associated with regulation of the migratory ability and survival in human glioma cells. These findings suggest novel mechanisms of malignancy and new potential combinatorial strategies for the management of malignant glioma.

## INTRODUCTION

Human tumors tend to contain a variety of cellular subpopulations with different characteristics and behaviors with respect to cellular activities involved in gene expression, morphology changes, metabolism, proliferation, drug responsiveness, and motility [1-3]; this property is geneally referred to as intratumoral heterogeneity. The cancer stem cell hypothesis is a prevailing model to explain intratumoral heterogeneity [4, 5]. Indeed, most human cancers contain distinct cellular subpopulations with different genetic alterations and behaviors [6]. Several studies have combined genetic and molecular approaches to evaluate the phenotypic differences between different grades of glioma [7]. For example, Cheng et al. [8] checked for the presence of cell subpopulations among U251 and U87 glioma cell by using a reproducible method for investigating the invasive behavior of cells. The use of a three-demensional experimental model for detecting changes in proliferation markers reflecting heterogeneity in glioma is a valuable approach toward drug development. The subpopulations identified in glioma showed differences with respect to their sensitivity toward chemotherapy agents, which was also observed by Shapiro et al. [9]. Furthermore, Xu et al. [10] pointed out that development of cell resistance to bevacizumab anti-angiogenic therapy might be due to upregulation of angiogenic pathways or genomic constitution changes. In our current study, we selected U251 sublines and investigated the heterogeneity of these different cell lines in the resulting tumor with respect to control of cell motility and survival. The property of intratumoral heterogeneity has long been associated with tumor metastatic potential and therapeutic resistance [11]. Indeed, the intratumoral heterogeneity of malignant glioma has been reported to be responsible for the high frequency of therapeutic failure [12, 13]. These findings indicate that it is important to understand the heterogeneity of glioma in order to develop more effective treatment strategies.

Micro-RNAs (miRNAs) are endogenous small non-coding RNAs of 20-25 nucleotides that target the 3′-untranslated region of target mRNAs to induce translational silencing and negatively regulate protein expression. MiRNAs play pivotal roles in a wide range of biological processes such as proliferation, apoptosis, metabolism, development, and differentiation [14]. MiRNA expression profiling in human cancers has revealed signatures that are closely associated with the diagnosis, staging, progression, prognosis, and responsiveness to therapies [15]. In particular, miR-21 has been shown to be overexpressed in several tumors, including glioma and breast cancer [16, 17]. miR-21 has been reported to regulate cellular contractility and the extracellular matrix composition, so that its overexpression leads to enhanced cell invasion and metastasis [18]. In addition, previous clinical studies demonstrated that increased miR-21 expression was positively associated with more advanced stages and poorer survival in kidney and colon cancer patients [19, 20], whereas treatment of antisense miR-21 to prostate cancer cells resulted in increased sensitivity to apoptosis and inhibition of cell invasion [21].

Curcumin, a phyto-polyphenolic pigment derived from turmeric (*Curcuma longa*), has been suggested as a potential anti-cancer agent for many tumor types [22]. It has been used to suppresses inflammation, induces apoptosis, reduces invasion and angiogenesis, and sensitizes tumor cells to therapeutic agents. Curcumin has been shown to inhibit tumor progression by modulating a large number of molecules and signal transduction pathways [23, 24]. A recent report showed that downregulation of miR-21 was linked to the anticancer functions of curcumin, and that restoring miR-21 expression antagonized curcumin-induced apoptosis [25]. Previous studies demonstrated that treating malignant glioblastoma cells with curcumin altered the expression of Bcl-2, pro-caspase-3, and pro-caspase-9, as well as the apoptosis pathway [26].

Mechanism by which miRNAs can regulate apoptosis is through the activation of caspases and death receptor (DR) signal transductions [27]. DR4 and DR5 induce apoptosis upon binding to their ligand, tumor necrosis factor-related apoptosis-inducing ligand (TRAIL). miR-21 was shown to inhibit TRAIL-dependent apoptosis by suppressing the expression of caspase-3 in glioma cells [28]. Moreover, suppression of miR-21 was shown to be involved in the Smoothened inhibitor-induced activation of DR4 or DR5, subsequently induces apoptosis, and reduced cell viability in glioblastoma [29].

In this study, we evaluated differences between parental glioma cells and selected migration-prone subline cells with respect to migration abilities, oncogenic miR-21 expression, and cytotoxic sensitivities. In particular, miR-21 was upregulated in migration-prone subline cells, and was found to regulate the expression of the Bcl-2 family and apoptosis-related proteins. Thus, understanding the role of miRNAs in regulating intratumoral heterogeneity will provide novel insights into the molecular basis of cancers, and might help to identify new biomarkers for cancer diagnosis and therapy.

## RESULTS

### The migration-prone sublines and parental cells exhibited differential migratory abilities and protein expression profiles

**Figure 1:**
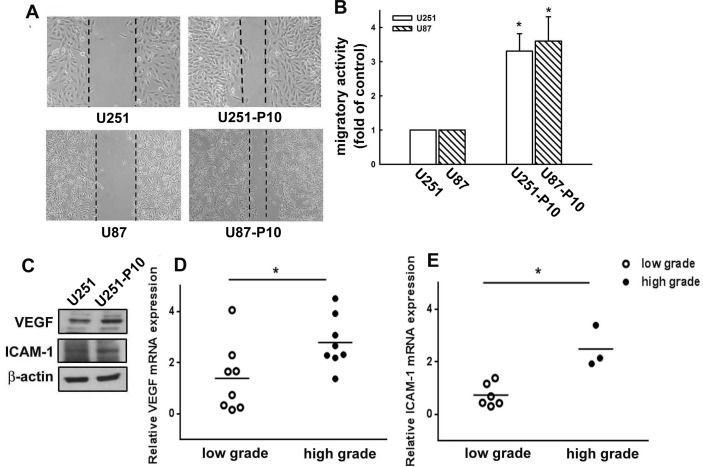
Migration-prone subline cells exhibit higher migratory ability than parental glioma cells **A.** After 10 rounds of selection, U251 or U87 and their corresponding migration-prone subline P10 cells were seeded for 24 h. Cell migration was determined using a wound-healing assay. Migration-prone subline cells showed faster healing ability than parental cells. **B.** *In vitro* migration activity was measured using a cell culture insert system 24 h after U251 or U87 cells and migration-prone subline P10 cells were seeded. Migrated cells were visualized using phase-contrast microscopy. U251-P10 and U87-P10 cells exhibited enhanced migration ability compared with parental cells. Representative images are shown. **C.** The protein expression profiles of U251 and U251-P10 cells. Protein expression levels of VEGF and ICAM-1 were determined using western blotting. **D.** Relative quantification of *VEGF* mRNA or **E.** I*CAM-1* mRNA in low-grade and high-grade brain tumors was determined by quantitative real-time PCR. Quantitative data are presented as mean ± SEM of three independent experiments.

The migration-prone subline cells were selected from two different glioma cell lines, U251 and U87, and designated as U251-P10 and U87-P10, respectively. The migratory activities of U251, U87, U251-P10, and U87-P10 cells were examined using wound-healing and transmigratory assays. In the wound-healing model, the U251-P10 and U87-P10cells exhibited enhanced healing ability compared with U251 and U87 cells (Figure 1A). As shown in Figure 1B, U251-P10 and U87-P10 cells also showed increased cell mobility and migrated more easily through the membrane pores of cell culture inserts compared with U251 and U87 cells, respectively. The mobility of the cells of migration-prone sublines was approximately 3.2- and 3.5-fold higher than that of U251 and U87 cells, respectively. Protein analysis showed that the expression levels of two invasive-associated molecules, VEGF and intracellular cell adhesion molecule 1 (ICAM-1), were higher in U251-P10 cells than in U251 cells (Figure 1C). We further investigated the *VEGF* and *ICAM-1* mRNA expression levels from samples of patients with low-grade and high-grade glioma. Real-time PCR showed a significantly higher level of *VEGF* mRNA in the high-grade samples compared with the-low grade samples (Figure 1D). In addition, a higher level of *ICAM-1* mRNA expression was also observed in glioma samples classified as high grade (Figure 1E). Our data indicated that up-regulation of VEGF and ICAM-1 is associated with the pathological features of gliomas migration. Thus, elevated expression of VEGF and ICAM-1 in migration-prone cells may be involved in the autocrine or paracrine functions that subsequently enhance migration.

### miR-21 regulates cell motility and the expression of apoptosis-related proteins

**Figure 2:**
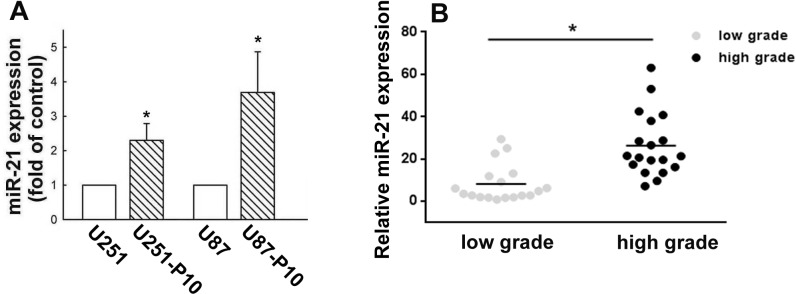
Elevated expression of miR-21 in cells of migration-prone sublines and high-grade glioma samples **A.** Quantitative real-time PCR for miR-21 was performed using a TaqMan microRNA Assay kit. Migration-prone subline P10 cells expressed more oncogenic miR-21 in both U251 and U87 cell lines. Quantitative data are presented as mean ± SEM of three independent experiments; *p < 0.05 compared with parental cells. **B.** Relative miR-21 expression in low-grade and high-grade gliomas was analyzed using quantitative real-time PCR. Quantitative data are presented as mean ± SEM, *p < 0.05 compared with low-grade gliomas.

**Figure 3:**
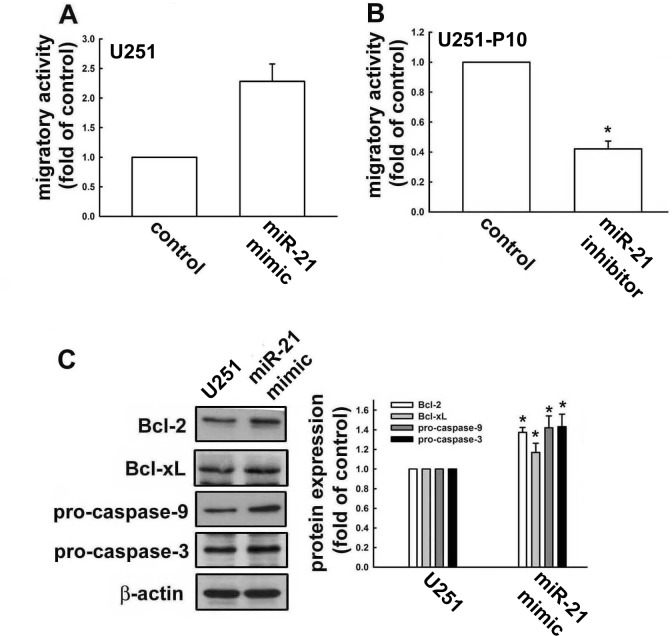
miR-21 expression is involved in regulation of apoptotic pathways and promotes cell migration **A.** *In vitro* migration activities were determined after U251 cells were transfected with negative control miRNA (NC) or miR-21 mimic, and **B.** U251-P10 cells were transfected with miR-21 inhibitor for 48 h. Results are representative of four independent experiments. Representative images are shown, and quantitative data are presented as mean ± SEM of three independent experiments; *p < 0.05 compared with U251 or U251-P10 cells. **C.** U251 cells were transfected with negative control miRNA (NC) or miR-21 mimic for 48 h. Cell lysates were analyzed by western blotting with antibodies against Bcl-2, Bcl-xL, pro-caspase-9, and pro-caspase-3. Representative images are shown, and quantitative data are presented as mean ± SEM of three independent experiments; *p < 0.05 compared with U251 cells.

miR-21 has been reported to be highly expressed in malignant tumors and to play a role in the regulation of cell migration. Therefore, we compared the miRNA and protein expression profiles between migration-prone subline cells and parental cells. For both U251 and U87 cells, the migration-prone subline cells showed higher expression levels of oncogenic miR-21 than the parental cells (Figure 2A). This same difference in miR-21 expression was also observed between low-grade and high-grade human glioma samples, in which miR-21 expression was significantly elevated in the high-grade glioma samples (Figure 2B). We further investigated the involvement of miR-21 in cell motility. As shown in Figure 3A, the U251 cells demonstrated a 2.5-fold increase in migration activity after being transfected with miR-21 mimic. Furthermore, transfection with an miR-21 inhibitor attenuated the migration activity of the migration-prone U251-P10 cells (Figure 3B). These data demonstrated a correlation between cell motility and oncogenic miR-21 expression. Moreover, the protein expression levels of Bcl-2, Bcl-xL, pro-caspase-9, and pro-caspase-3 were upregulated in U251-P10 cells compared to U251 cells (Supplementary Figure 1). We then assessed the correlation of the expression of these proteins with miR-21 expression. U251 cells were transfected with either a miRNA negative control or miR-21 mimic. The expression levels of anti-apoptotic proteins such as Bcl-2, Bcl-xL, pro-caspase-9, and pro-caspase-3 were upregulated after transfection with the miR-21 mimic in U251 cells (Figure 3C). Collectively, these results, combined with the elevated miR-21 expression in migration-prone subline cells and high-grade human glioma samples, indicated that miR-21 may play an important role in cancer progression.

### Migration-prone subline cells showed lower sensitivity to curcumin-induced cell death

**Figure 4:**
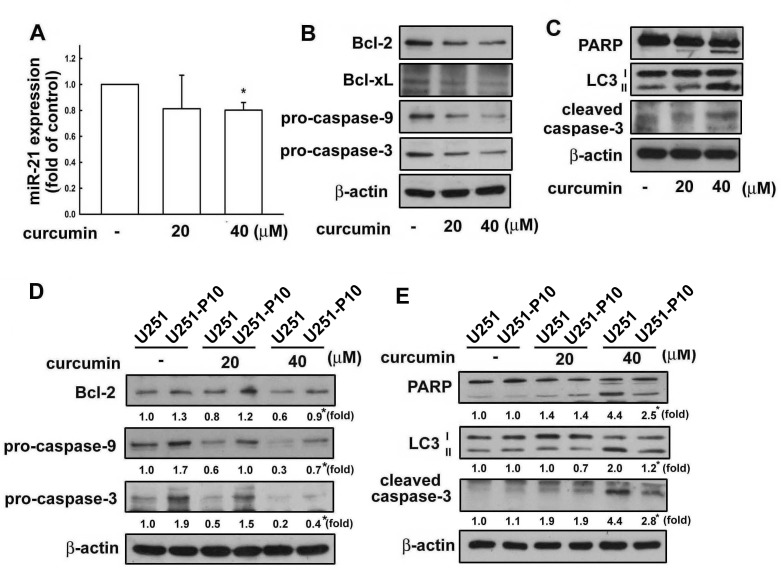
Curcumin regulates the expression of apoptosis-associated proteins in U251 cells **A.** U251 cells were treated with different concentrations (20 or 40 μM) of curcumin for 8 h, and the expression of miR-21 was determined using quantitative real time-PCR. **B.** U251 cells were treated with different concentrations (20 or 40 μM) of curcumin for 24 h, and the expression of Bcl-2, Bcl-xL, pro-caspase-9, pro-caspase-3, **C.** LC3-II, and cleaved PARP and caspase-3 was determined using western blotting. **D.** U251 and U251-P10 cells were treated with curcumin (20 or 40 μM) for 24 h, and the expression of Bcl-2, Bcl-xL, pro-caspase-9, pro-caspase-3, **E.** LC3-II, and cleaved PARP and caspase-3 was also determined using western blotting. Representative images are shown, and quantitative data are presented as mean ± SEM of three independent experiments; *p < 0.05 compared with U251 cells.

miRNAs are important molecules in cancer initiation and progression. As described above, the heterogeneity between U251 and U251-P10 cells could be attirbuted to differential expression of oncogenic miR-21. Therefore, we next examined the effect of the anti-cancer drug curcumin on miR-21 expression in these cell lines. The expression of miR-21 was down-regulated in cells treated with curcumin (Figure 4A). As shown in Figure 4B, curcumin treatment to U251 cells also resulted in reduced expression of Bcl-2, Bcl-xL, pro-caspase-9, and pro-caspase-3 in a dose-dependent manner. Conversely, the expression levels of microtubule-associated protein light chain 3 (LC3-)I/II and the cleaved forms of PARP and caspase-3 proteins were up-regulated after curcumin treatment, indicating that curcumin induces a pro-apoptosis pathway (Figure 4C). Furthermore, the protein expression levels of Bcl-2, pro-caspase-9, and pro-caspase-3 were all increased in U251-P10 cells when compared with U251 cells (Figure 4D), and this difference was maintained even in the presence of 20 μM or 40 μM curcumin (note the asterisk in Lane). In addition, U251-P10 cells showed reduced expression of LC3-II and the cleaved forms of PARP and caspase-3 after treatment with curcumin. These findings suggest that cells of the migration-prone subline U251-P10 had lower sensitivity to curcumin-induced cell death than U251 cells.

### Migration-prone subline cells exhibited lower cytotoxic sensitivities to curcumin-induced cell death through DR

**Figure 5:**
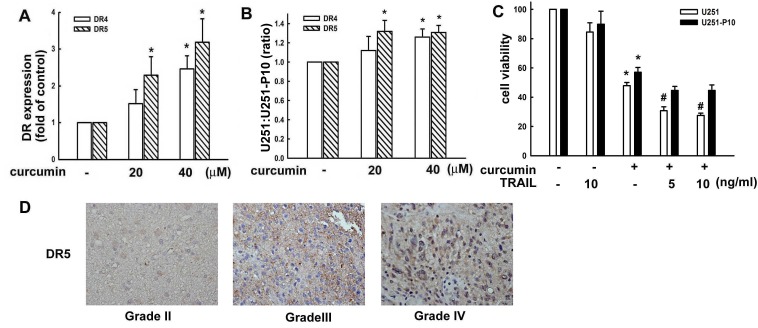
Migration-prone subline cells have a lower sensitivity to curcumin-induced cell death through death receptors **A.** U251 cells were treated with different concentrations (10, 20, or 40 μM) of curcumin for 8 h, and DR4 and DR5 expression was analyzed by flow cytometry. **B.** U251 and U251-P10 cells were treated with different concentrations (20 or 40 μM) of curcumin for 8 h, and DR4 or DR5 expression was analyzed by flow cytometry. DR4 and DR5 in expression was increased in U251 cells compared with U251-P10 cells, leading to an enhanced U251:U251-P10 cell ratio. **C.** U251 and U251-P10 cells were pretreated with different concentrations (5 or 10 ng/mL) of TRAIL and were then treated with curcumin for 24 h, and cell viability was determined by the MTT assay. The quantitative data are presented as mean ± SEM of three independent experiments; *p < 0.05 compared with parental cells. # *p* < 0.05 compared with the curcumin treatment group. **D.** Representative immunohistochemical images of DR5 expression in paraffin-embedded sections of malignant glioma specimens. DR5 expression in malignant gliomas of different grades according to the WHO classification system was analyzed by immunohistochemistry.

We next evaluated the responses of U251 and U251-P10 cells to induction of extrinsic cell death signals. As shown in Figure 5A, flow cytometry revealed that the expression of both DR4 and DR5 was enhanced by curcumin treatment in a dose-dependent manner in U251 cells. In addition, curcumin induced the expression of DR4 and DR5 to a greater extent in U251 than in U251-P10 cells, leading to an increased ratio of U251:U251-P10 cells (Figure 5B). The activation of DR pathways plays a critical role in a variety of drug-induced apoptotic cell death pathways. Therefore, we further studied whether the variation in curcumin induced cell death rates between U251 and U251-P10 cells might be attributed to differences in the activation of DR pathways. As shown in Figure 5C, curcumin treatment reduced cell viability in both U251 and U251-P10 cells. Interestingly, curcumin was more cytotoxic to U251 cells than to U251-P10 cells, as determined with MTT assays. Moreover, U251 cells that were pretreated with TRAIL showed significantly elevated curcumin-induced cell death, whereas this augmentation of curcumin-induced cell death was not statistically significant in U251-P10 cells (Figure 5C). These results, along with the data shown in Figure 4, indicated differences between U251 and U251-P10 subline cells with respect to the response to the induction of cell death in both the intrinsic pathway and extrinsic pathway. In addition, the DR expression level differed among various grades of malignant human glioma specimens. As shown in Figure 5D, DR expression showed an increased tendency in human glioma tissue specimens at more advanced grades (i.e., grades III and IV).

## DISCUSSION

Previous studies have shown that miR-21 expression is higher in malignant cells and tumor microenvironment components compared with those of adjacent non-tumor tissues [30]. Dysregulated miR-21 expression was observed in glioblastoma samples and tumor-associated blood vessels compared with non-neoplastic brain tissue [14, 31]. Furthermore, clinical reports revealed that elevated miR-21 expression correlated with tumor grade and was associated with poorer overall survival in glioma patients [32]. Moreover, miR-21 was demonstrated to act as a key regulator of multiple pathways that mediate cancer cell proliferation [33] and survival [34]. For example, miR-21 overexpression contributes to the reduced expression of apoptosis-related genes [35]. In this study, we demonstrated that miR-21 expression is correlated with the migration abilities as well as cytotoxic sensitivities of glioma cells. These results indicate the potential value of miR-21 as a biomarker for therapy and a prognostic factor for glioma patients, which may contribute to improved assessment of survival probability and treatments. Thus, our study on the functional difference of gliomas provides a new perspective for the development of prognostic biomarkers to anticipate cancer progression. Furthermore, the correlation between miR-21 expression and cell motility mediatingcould provide insight into the association of diverse protein expression profiles with different clinicopathological parameters in gliomas.

The present study indicates that curcumin sensitizes glioma cells to TRAIL-induced apoptosis through activating both the extrinsic and intrinsic apoptosis pathways. Accumulating evidence suggests that knockdown of miR-21 triggers caspase activation, leading to increased cell death and inhibition of tumor cell growth by modulating the expression of apoptosis-associated proteins [36, 37]. It is believed that the heterogeneity of a tumor might contribute to its ability to overcome the host defense response against invasion and metastasis, leading to the development of resistance to anticancer drugs [38]. A previous report showed that inhibition of miR-21 expression decreased matrix metalloproteinase (MMP) activities, which reduced the motility and invasion of glioma cells [39]. MiR-21 is also known to contribute to glioma's resistance to chemotherapy by targeting tumor suppressor genes such as *FBXO11* and *PDCD4* [40-42]. Several studies have documented a role for miR-21 in protecting glioma cells from chemotherapy drugs *via* regulation of Bcl-2 and caspase-3 activity [43, 44]. For example, Cheng et al. [8] evaluated the differences between spheroids generated from two different cell lines in terms of their proliferative activities and the caspase expression. Subpopulations of cells existing in the spheroid cores were identified, which appeared to have a survival advantage in response to treatment. This suggests that high levels of miR-21 and anti-apoptosis proteins are important factors for the regulation of chemosensitivity in glioma.

A few studies have investigated the prognostic value of DR4 and DR5 expression levels in other tumor types [45, 46]. The DR5 expression level decreased as clinical staging progressed in patients with laryngeal squamous cell carcinoma, but an increase in DR5 expression was associated with advanced clinical stage in oral cavity squamous cell carcinoma [47]. Stronger DR5 tumor staining was associated with an adverse prognosis in non-small-cell lung cancer [48] and breast cancer [49]. Malignant glioma cells have been found to mainly express DR5, and an inverse relationship between the *DR4* mRNA expression level and WHO grade has been reported in various astrocytic tumors [50]. Indeed, Jos et al. [50] stated that DR4 and DR5 expression in cell lines cannot accurately reflect the heterogeneous phenotype of primary GBM tissue. Rieger et al. [51] reported that induced DR expression may be a response of the defense system of glioma cells to mediate tumor immune escape. However, DR expression may also have a decisive role in determining TRAIL-induced DR-mediated apoptotic signaling [52, 53]. In this study, we showed that human glioma cells express DR4 and DR5 proteins (Supplementary Figure 2A) and that malignant human glioma specimens express DR5 (Figure 5D). Moreover, clinical studies determined that treatment with conatumumab (an anti-DR5 agent) in breast cancer patients could be considered safe [54], and improved the survival rate in phase II studies of colorectal cancer patients [55]. Therefore, these profiles of DRs and their ligands could be important for predicting the responsiveness of malignant gliomas to treatment with TRAIL and chemotherapy drugs in future clinical trials.

Tumor cell migration is essential for invasion and dissemination into surrounding tissues. Growth factors and other chemotactic agents can mediate cell migration and proliferation during tumorigenesis. We previously established a cancer cell model with high migratory ability, and revealed that the migration-prone sublines showed both higher migration and invasion activity [56, 57]. We found more prominent expression of glial cell line-derived neurotrophic factor (GDNF) in migration-prone cells, which also showed higher levels of MMP-13 expression [58]. In the current study, we further confirmed that after 10 rounds of selection, the migration-prone U251-P10 cells expressed higher levels of MMP-13 (Supplementary Figure 2B). We also previously performed *in vivo* glioma xenograft experiments, and demonstrated that U251 cells inoculated in the brains of nude mice grew as noninvasive solid tumor masses (ball-like) with negligible localized invasion and a small tumor size. In contrast, the cells of the migration-prone subline U251-P10 grew in the mouse brains with a diffuse tumor boundary and fingerlike protrusions [57]. Thus, the present *in vitro* experiments confirmed these *in vivo* findings, suggesting that migration-prone subline cells show increased migratory activity compared with parental cells.

In summary, this study identified functional differences between migration-prone subline cells and parental cells. miR-21 expression was upregulated in both the migration-prone cells as well as in samples from high-grade glioma patients. Furthermore, the expression levels of the migration-related molecules VEGF and ICAM-1 were also increased in malignant gliomas. Enforced expression of miR-21 in parental cells *via* transfection with an miR-21 mimic restored the expression of the anti-apoptotic proteins pro-caspase-9 and pro-caspase-3. Taken together, these findings demonstrate that altering the expression of oncogenic miR-21 and migration- and apoptosis-related proteins could restore curcumin-induced cell death. These observations suggest that the extent of intratumoral heterogeneity in glioma influences cell migratory activity as well as the expression of intrinsic and extrinsic apoptotic proteins. Therefore, altering the migratory activity and regulating the levels of intratumoral heterogeneity might be a potential strategy for treating glioma. Further functional studies on potential molecular markers may provide novel therapeutic strategies for specifically targeting cellular invasion and metastasis to effectively treat malignant gliomas.

## MATERIALS AND METHODS

### Materials

Primary antibodies specific for Bcl-2, Bcl-xL, PARP, pro-caspase-3, pro-caspase-9, and β-actin were obtained from Santa Cruz Biotechnology (Santa Cruz, CA, USA). Primary antibodies were also obtained against cleaved caspase-3 (Cell Signaling Technology, Danvers, MA, USA), DR4 and DR5 (Imgenex, San Diego, CA, USA), and LC3-I/II (Novus Biologicals, Littleton, CO, USA). Curcumin was purchased from Sigma-Aldrich (St. Louis, MO, USA). The miRNA negative control, and miR-21 mimics and inhibitors were acquired from Ambion Life Technologies (Carlsbad, CA, USA).

### Cell culture

U251 and U87 human brain glioma cells were obtained from the American Type Culture Collection (Manassas, VA, USA) and maintained in 75-cm^2^ flasks in Dulbecco's modified Eagle medium (DMEM). All cells were cultured in DMEM supplemented with 10% FBS, 100 U/mL penicillin, and 100 mg/mL streptomycin, and were incubated at 37°C in a humidified atmosphere containing 5% CO_2_ and 95% air.

### Patients and specimen preparation

The study was performed following the guidelines of the Institutional Review Board of Chang Gung Memorial Hospital, and all subjects provided informed written consent before their enrollment. The tumor tissue specimens were acquired from patients who had been diagnosed with glioma and had undergone surgical resection at Chang Gung Memorial Hospital. The pathological grades (on a scale of I to IV) of each glioma specimen were verified through histological examination between February 2005 and November 2012 by a neuropathologist according to the World Health Organization criteria. The tissue samples for miRNA or mRNA examination were sharply excised, placed in sterile tubes, and frozen immediately in liquid nitrogen. The samples were stored at −80°C until analysis.

### Immunohistochemistry

The protocol for immunohistochemistry was performed as described in our previous report [59]. Briefly, tissue specimens on glass slides were rehydrated and quenched for endogenous peroxidases with hydrogen peroxide. After being deparaffinized, the sections were blocked by incubation in PBS containing 3% bovine serum albumin. The anti-DR5 primary antibody was applied to the slides at a dilution of 1:50, followed by overnight incubation at 4°C. After several washes with PBS, the samples were incubated with the biotin-conjugated secondary antibody at a dilution of 1:50. Bound antibodies were detected with the ABC reaction kit (Vector Laboratories; Burlington, CA, USA), developed with diaminobenzene (Sigma-Aldrich), and counterstained with hematoxylin.

### Transmigratory assay

A transmigratory activity assay was performed as described previously [59, 60]. Briefly, cells in serum-free medium were seeded onto the upper chambers with 8-μm pore size polycarbonate filters (Corning, NY, USA). The cells were then incubated for 24 h at 37°C in a humidified incubator. Non-migrated cells remaining on the upper surface of the filters were removed by wiping with a cotton swab. Cells that penetrated through the pores and migrated to the underside of the filters were stained with 0.05% crystal violet solution containing 20% methanol. The number of stained cells was counted in three random fields per well under a light microscope and images of the migrated cells were acquired with a digital camera.

### Wound-healing assay

U251 or U87 glioma cells were seeded onto cell culture plates with Ibidi Culture-Inserts in the middle of the wells. A cell-free gap of 500 μm was thus generated after removing the Ibidi Culture-Insert. The images were acquired after 0, 8, and 24 h using a digital camera and a light microscope.

### Establishment of the migration-prone sublines

Subpopulations of glioma cells were selected according to their differential migration ability using the previously described cell culture insert system [61]. After 24 h of migration, cells that had penetrated through the pores and migrated to the underside of the filters were trypsinized, harvested and cultured for 2 days for a second round of selection. After 10 rounds of selection, the subline was designated as the migration-prone subline of U251-P10 or U87-P10 cells.

### RNA extraction and quantitative real-time PCR

RNA was extracted from glioma cells using TRIzol Reagent (Invitrogen, Carlsbad, CA, USA) and was quantified using the Biodrop spectrophotometer (Cambridge, UK). The expression levels of miR-21 were detected using quantitative real time-PCR and the TaqMan MicroRNA Assay kit (Applied Biosystems, Foster City, CA, USA) as described in the manufacturer protocols. Two micrograms of total RNA from each sample was reverse-transcribed using the TaqMan MicroRNA Reverse Transcription kit (Applied Biosystems). PCR amplifications were performed in final volumes of 20 μL using TaqMan Universal PCR Master mix. The amplifications were initiated by incubation at 95°C for 10 min followed by 40 cycles of 95°C for 15 s and 60°C for 60 s. The expression of U6 RNA was used as an internal control to normalize the expression levels of miRNAs.

### Western blotting

Proteins were extracted from samples as described previously [62] resolved by electrophoresis, and transferred to polyvinylidene difluoride membranes (Millipore, Bedford, MA, USA). The membranes were incubated for 1 h with 4% dry skim milk in PBS to block non-specific bindings, followed by incubation overnight at 4°C with primary antibodies. After three washes in PBS with Tween-20, the membranes were incubated with peroxidase-conjugated secondary antibodies (Santa Cruz Biotechnology) for 1 h at room temperature. The blots were visualized by enhanced chemiluminescence using Kodak X-OMAT LS films (Rochester, NY, USA).

### Transfection

Glioma cells were transiently transfected with either miR-21 mimic, miRNA negative control, or the inhibitor (Ambion) using Lipofectamine 2000 (LF2000; Invitrogen) for 48 h. MiR-21 mimic, miRNA negative control, or inhibitor were pre-mixed with LF2000 in OPTI medium (Invitrogen) for 20 min before application to the cells. An equal volume of medium containing 10% FBS was added 4−6 h later. After 24 h of incubation, LF2000-containing media were replaced with fresh serum-free media.

### Flow cytometry

Glioma cells were cultured in six-well plates. Cells were treated with vehicle or curcumin for 8 h, washed with PBS, and trypsinized at 37°C. The cells were fixed for 10 min in 4% paraformaldehyde and then rinsed with PBS followed by incubation with anti-DR4 or -DR5 antibodies for 1 h. After a brief wash, the cells were incubated with fluorescein isothiocyanate-conjugated secondary antibody for 1 h and analyzed by flow cytometry using FACSCalibur with CellQuest software (BD Biosciences).

### Cell viability assay

Cell viability was determined using an MTT assay. Cells on a 96-well plate were treated with curcumin for 24 h. The media were removed and the cells were washed with PBS followed by incubation with MTT (0.5 mg/mL) for 2 h at 37°C. The MTT reagent was then replaced with dimethyl sulfoxide (100 μL per well) to dissolve the formazan crystals. This process was facilitated by mixing the samples on a shaker at room temperature for 10 min, and then the absorbance at 550 nm was determined using a microplate reader (Bio-Tek, Winooski, VT).

### Statistical analysis

Statistical analysis was performed using the software Graphpad Prism 4.01 (GraphPad Software Inc., San Diego, CA, USA). The values are presented as mean ± SEM. The significance levels of differences between the experimental group and control groups were assessed using the Student's *t*-test. Statistical comparisons of more than two groups were performed using one-way analysis of variance with the Bonferroni post-hoc test. The difference was considered significant if the *p*-value was < 0.05.

## SUPPLEMENTARY MATERIAL FIGURES


