# Supplementary material for: Migration-prone glioma cells show curcumin resistance associated with enhanced expression of miR-21 and invasion/anti-apoptosis-related proteins
Source: Oncotarget. 2015 Oct 12;6(35):37770–81. doi: 10.18632/oncotarget.6092 (PMC4741964; doi:10.18632/oncotarget.6092)
Supplement: Supplementary file 1 [file oncotarget-06-37770-s001.pdf]

Migration-prone glioma cells show curcumin resistance associated with enhanced expression of miR-21 and invasion/anti-apoptosis-related proteins

Supplementary Material

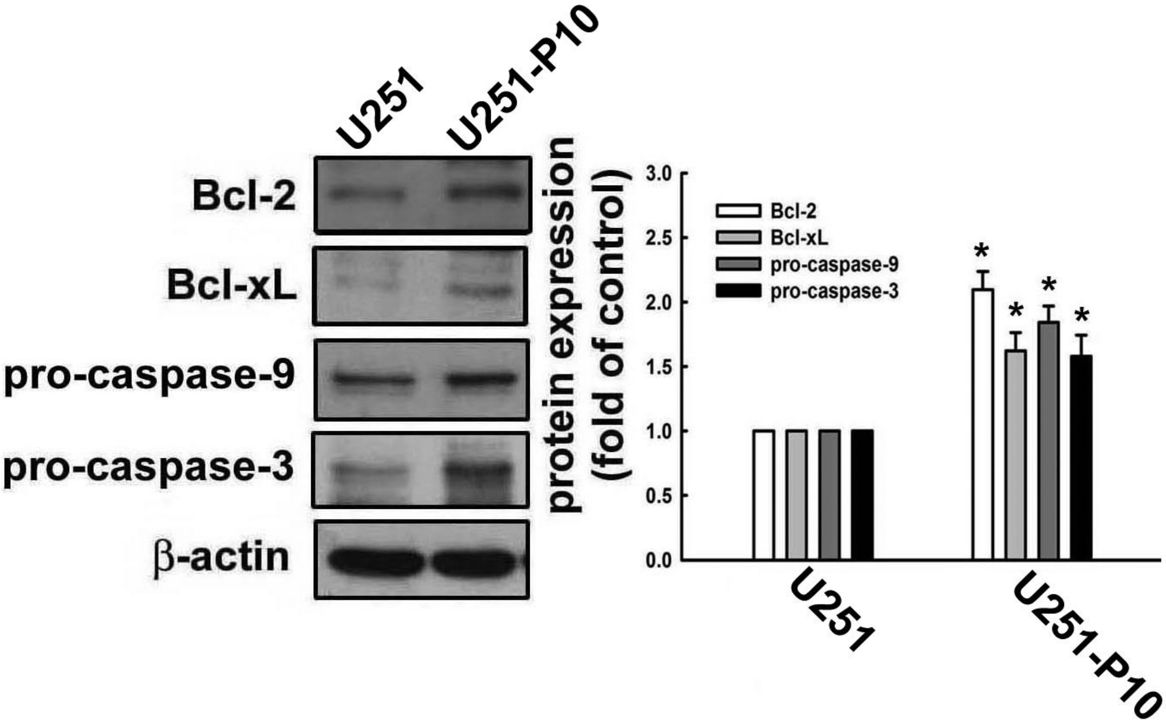

Supplementary Figure 1: Upregulation of anti-apoptosis and pro-caspase protein expression in migration-prone cells. The cell lysates of U251-P10 and the parental U251 cells were collected after 24 h of culture, and Bcl-2, Bcl-xL, pro-caspase-9, and pro-caspase-3 protein levels were determined using western blot analysis. Note that U251-P10 cells showed higher protein expression levels of Bcl-2, Bcl-xL, pro-caspase-9, and pro-caspase-3. Representative images are shown, and quantitative data are presented as mean ± SEM of three independent experiments; *p < 0.05 compared with U251 cells.

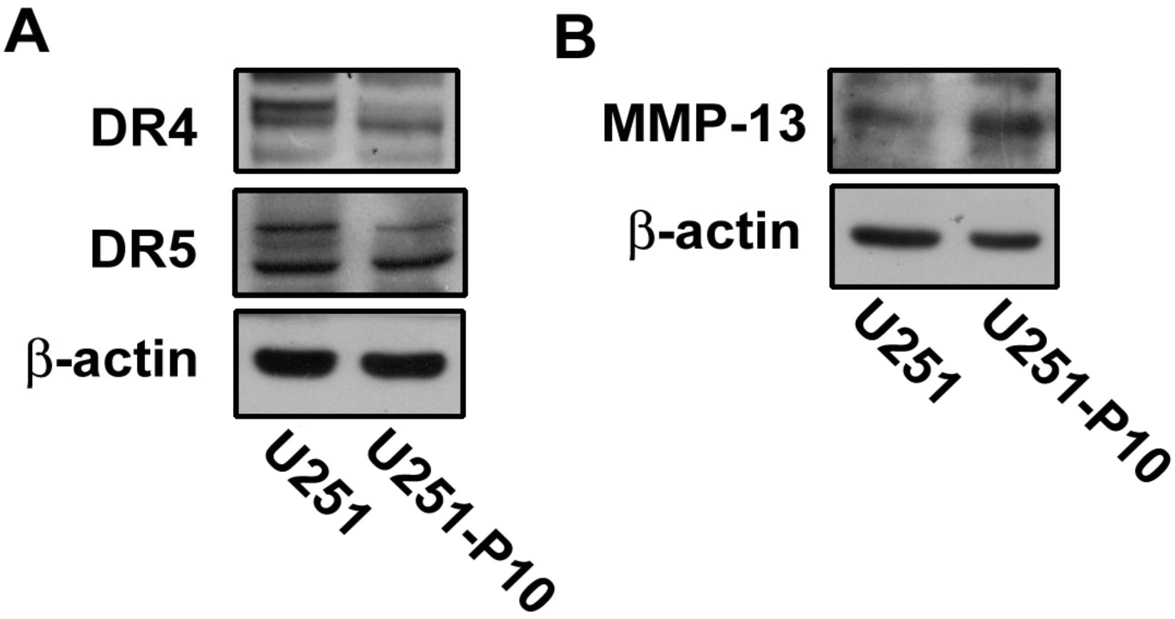

Supplementary Figure 2: The protein expression levels of DR and MMP-13 in U251 and U251-P10 cells. (A) The cell lysates of U251-P10 and the parental U251 cells were collected after 24 h of culture, and the expression levels of DR4 and DR5 were determined using western blotting. U251-P10 cells showed lower protein expression of DR4 and DR5 compared to U251 cells. The results are representative of at least three independent experiments. **(B)** U251 and U251-P10 cell lysates were analyzed by western blotting with antibodies against MMP-13. U251-P10 cells showed higher MMP-13 protein expression levels compared to the parental U251 cells. Similar results were obtained from three independent experiments.
